# Prosthetic nipple areola complex reconstruction after breast reduction: a case report

**DOI:** 10.1080/23320885.2026.2648923

**Published:** 2026-03-31

**Authors:** Salvatore Taglialatela Scafati, Fabrizio Schonauer, Dario d’Angelo, Francesco D’Andrea, Annachiara Cavaliere

**Affiliations:** ^a^ Plastiké STP, Plastic Surgery Clinic, Giugliano in Campania (Naples), Italy; ^b^ Plastic Surgery Unit, Federico II University Hospital, Naples, Italy; ^c^ Department of Emergency and Reception, Plastic Surgery Operative Unit, Hospital Center A. Cardarelli, Naples, Italy

**Keywords:** Breast reduction, nipple reconstruction, nipple implant

## Abstract

Total nipple-areola necrosis following breast reduction is a rare and unfortunate event. Several causes may be responsible for it, including compression of the vascular pedicle, venous congestion, infection, hematoma, or inappropriate surgical technique. Considering the importance of the nipple areola complex (NAC) in the overall appearance of a breast mound, it is clear that the complete loss of the nipple areola complex is one of the most traumatizing complications for both the patient and the surgeon. Herein we describe the case of a patient who developed complete NAC necrosis following breast reduction. After initial wound management and stabilization, the patient underwent delayed reconstruction using an innovative NAC implant specifically designed to restore nipple projection and contour. Delayed reconstruction of the left NAC was performed using a silicone implant (FixNip, GC Aesthetics, Caesarea, Israel), specifically designed to restore natural projection and contour. The implant was positioned under local anesthesia as an outpatient procedure. Postoperative course was uneventful. At 8 months, after ultrasound confirmed proper implant integration, medical tattooing of the areola was completed. Implant based NAC reconstruction achieved a stable and well-projected NAC reconstruction. No minor or major complications occurred, and the patient reported high satisfaction with the aesthetic result and psychological benefit from restoration of breast harmony. To our knowledge, this is the first described case demonstrating a successful implant-based NAC reconstruction following total necrosis after breast reduction. The FixNip implant, combined with medical tattooing, provided a safe and reliable solution with excellent cosmetic outcomes.

## Introduction

Breast hypertrophy is a condition characterized by very enlarged breasts, disproportionate to the woman’s figure. This body imbalance may cause physical and psychological impairment [1,2]. Breast hypertrophy is often associated to postural changes, shoulder and spinal pain leading to functional limitations.

Reduction mammaplasty aims to decreasing the breast size in order to restore the correct proportions of the female chest, providing also substantial pain relief and significant reduction of functional symptoms [3,4].

Complications related to breast reduction are similar to those associated with other breast procedures (hematoma, seroma, infection, delayed wound healing). However, some complications like nipple areola complex (NAC) necrosis, hypertrophic scarring and breast mound asymmetries are specifically related to breast reduction [5].

Partial or complete loss of the NAC is perhaps the most devastating potential complication associated with breast reduction [6].

BMI, smoking history, and higher resection weight have a significant relationship with NAC necrosis. Also, the pedicle choice has proven to influence the risk of nipple necrosis, ranging from 0.8 to 2.3% for the inferior pedicle, 2.1% for the superomedial pedicle and 2.3% for the superolateral pedicle technique [7]

In case of partial or complete loss of the NAC, conservative wound management with debridement and secondary healing may be indicated, while standard reconstructive techniques of NAC can be performed in a second stage [6].

The cosmetic success of NAC reconstruction depends by position, shape, texture, pigmentation, and especially projection. Countless techniques have been reported in the literature, including medical 3D tattoos, local flap, free tissue transplantation, and local flap combined with autologous or allogeneic transplantation, nevertheless no gold standard has emerged as a consistently reliable method of nipple reconstruction [8].

Long-term loss of nipple projection is the main concern burdening most of the reconstruction techniques and is the main cause of patient dissatisfaction [9,10].

Several studies comparing the most frequently used flap-based reconstructions reported that nipple projection is considered stable at 12 months after reconstruction and that the loss of projection during this period ranged from 40% to 70% [11].

Tissue engineered scaffolds printed in 3D have been recently proposed for nipple reconstruction. However, these techniques are still considered more experimental than practical and are not available in common clinical practice.

Herein we report the first case of a total nipple areola complex reconstruction following complete necrosis after breast reduction using an innovative NAC implant [12].

## Patients and methods

In November 2022 a 28 years old patient was scheduled for bilateral breast reduction in a private practice setting. Patient complained about her breasts size and lamented physical and psychological impairment related to her condition.

Preoperative physical examination showed bilateral macromastia with a grade III ptosis. NACs were pale and enlarged. Sternal notch- nipple distance was 31 cm on the right side and 31,5 on the left side.

**Figure 1.**
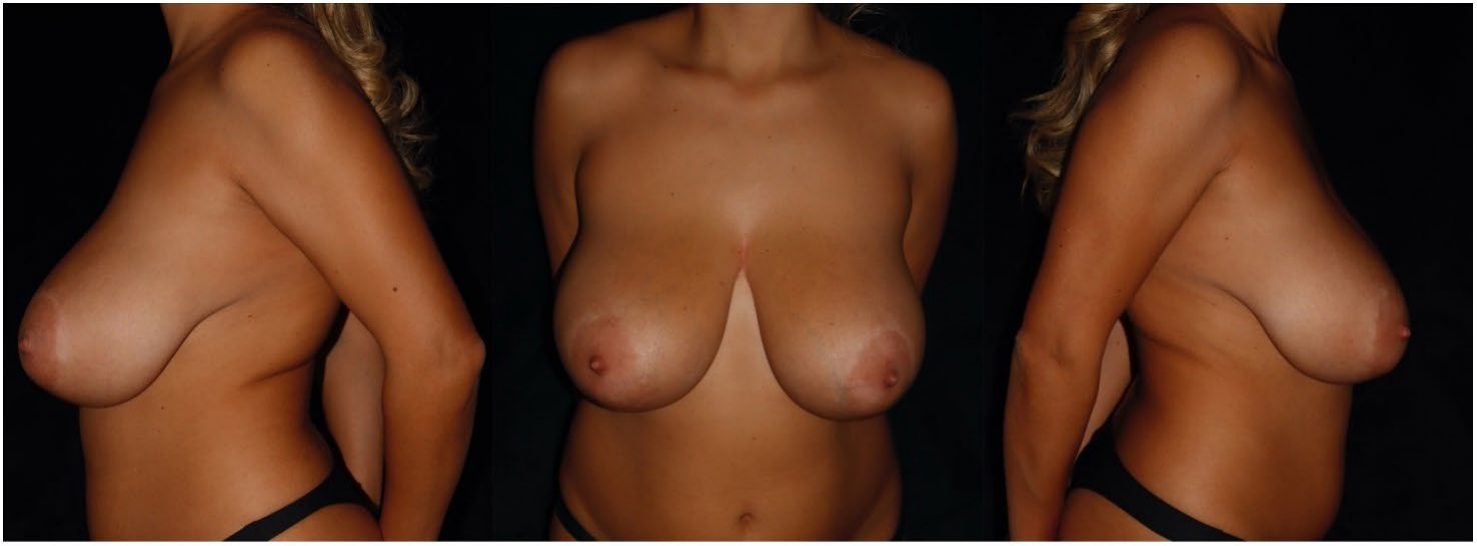
Preoperative photos (lateral and frontal view).

Routinely performed preoperative exams were negative for any concerning condition. Patient did not report any other health issue, drugs intake or previous surgeries. She was not a smoker, while BMI was 27. Patient had no history of pregnancies (Figure 1).

Patients underwent a bilateral breast reduction with inverted ‘T’ scar pattern. Standard surgical technique was performed. The NAC bearing pedicle was superomedial based. Approximatively 380 g were removed from the right side and 400 g from the left side. NACs were repositioned at 21 cm along the sternal notch-nipple axis, without tension and sutured in layers. Two Blake drains of 19 Fr were positioned. Surgical time was approximately 150 min.

At the end of the surgery, standard dressing was applied and postoperative compressive bra was worn by the patient. Mechanical thromboprophylaxis with anti-embolism stockings was used and maintained within seven days. During the first 24 h after surgery, signs of venous congestion started to appear on the left NAC. There were no signs of hematoma or infection such as erythema, fever, purulent discharge, or laboratory abnormalities.

Conservative treatment, including periareolar suture release and heparin-based ointment was immediately started. Unfortunately, after 10 days, a complete loss of the NAC was evident. Patient underwent 8 sessions of hyperbaric oxygen treatment while local wound care was prolonged to achieve definitive secondary intension closure.

**Figure 2.**
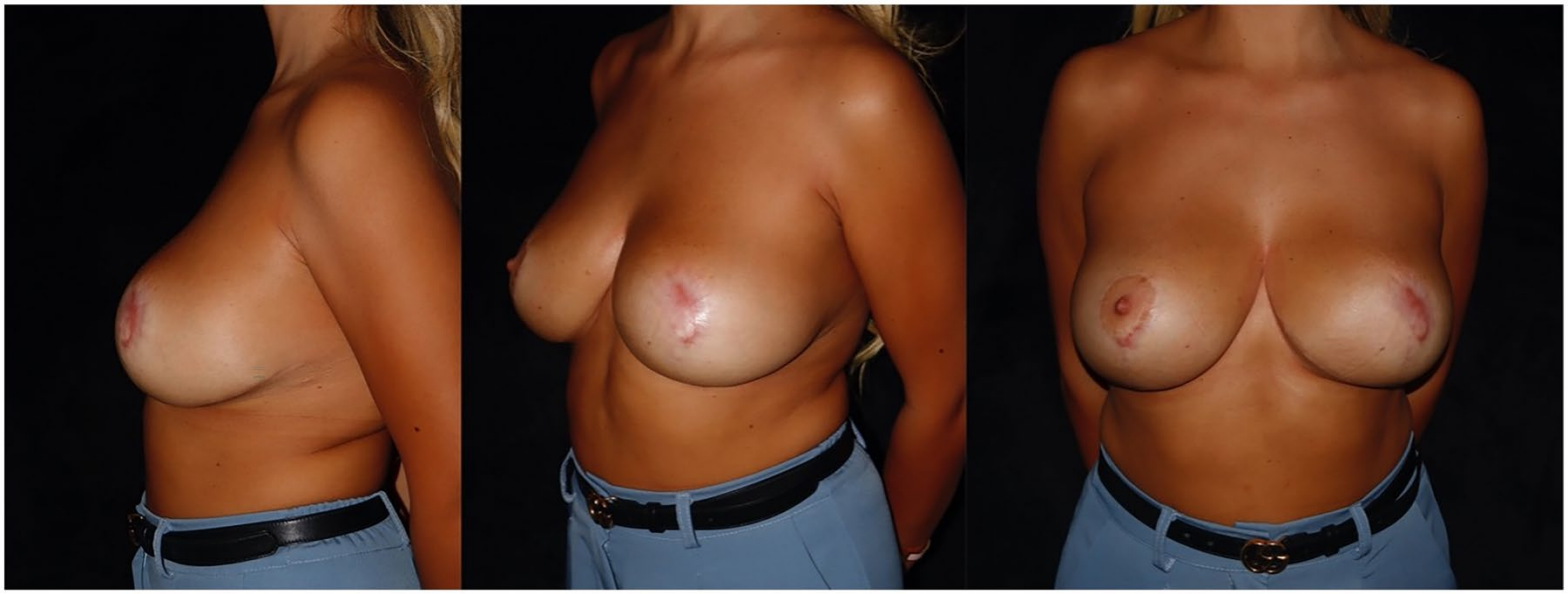
Postoperative photos showing the appearance of the scar after two session of nanofat graft, performed after 3 and 6 months respectively to improve the scar tissue left by secondary healing.

Two sessions of subcutaneous and intradermal nanofat were performed at 3 and 6 months following secondary wound healing to improve skin quality and soft tissue pliability (Figure 2).

At one year, a round shaped scar of 5 × 4,6 cm was present in correspondence of where the nipple should have been positioned. The scar was not hypertrophic or keloid. In October 2023, the NAC implant was positioned in an outpatient setting. The silicone implant (FixNip, GC Aesthetics, Caesarea, Israel) has been designed for aesthetic reconstruction of the female nipple in order to achieve a natural appearance with good nipple projection and limited prosthetic contours palpability and visibility. The implant includes a nitinol frame designed to provide a mechanical support fully covered by the silicone. The FixNip prosthesis is CE marked and ISO13485 certified, including compliance with European and international standards for medical devices [12].

With the patient standing upright in front of a mirror, the new left nipple areola complex position was marked in a symmetric position to the contralateral one using a circular areolar marker. The marking for implant placement was located partially beneath the pre-existing scar, corresponding to approximately the lateral half of the areola. Owing to prior lipofilling sessions, the scarred region was pliable and of adequate thickness thus ensuring appropriate prosthesis coverage. Specific written informed consent was obtained from the patient. According to institutional and national guidelines, ethical approval was not necessary for this case report, which is based on routine clinical practice and involves no experimental intervention. Under local anesthesia, a small 2 cm incision was made at the inferior border of the marked areolar perimeter. Subcutaneous dissection of the tissues underlying the marked areolar perimeter was performed to create a pocket which was irrigated with triple antibiotic solution with bacitracin, gentamicin and cefazolin. Finally, the nipple-areola implant was inserted and the pocket closed in layers. Standard dressing was applied and weekly visits were scheduled. Postoperative therapy including oral Cefixime 400 mg daily and on demand Paracetamol 1 g was given. Postoperative course was uneventful and no minor or major complications occurred.

**Figure 3.**
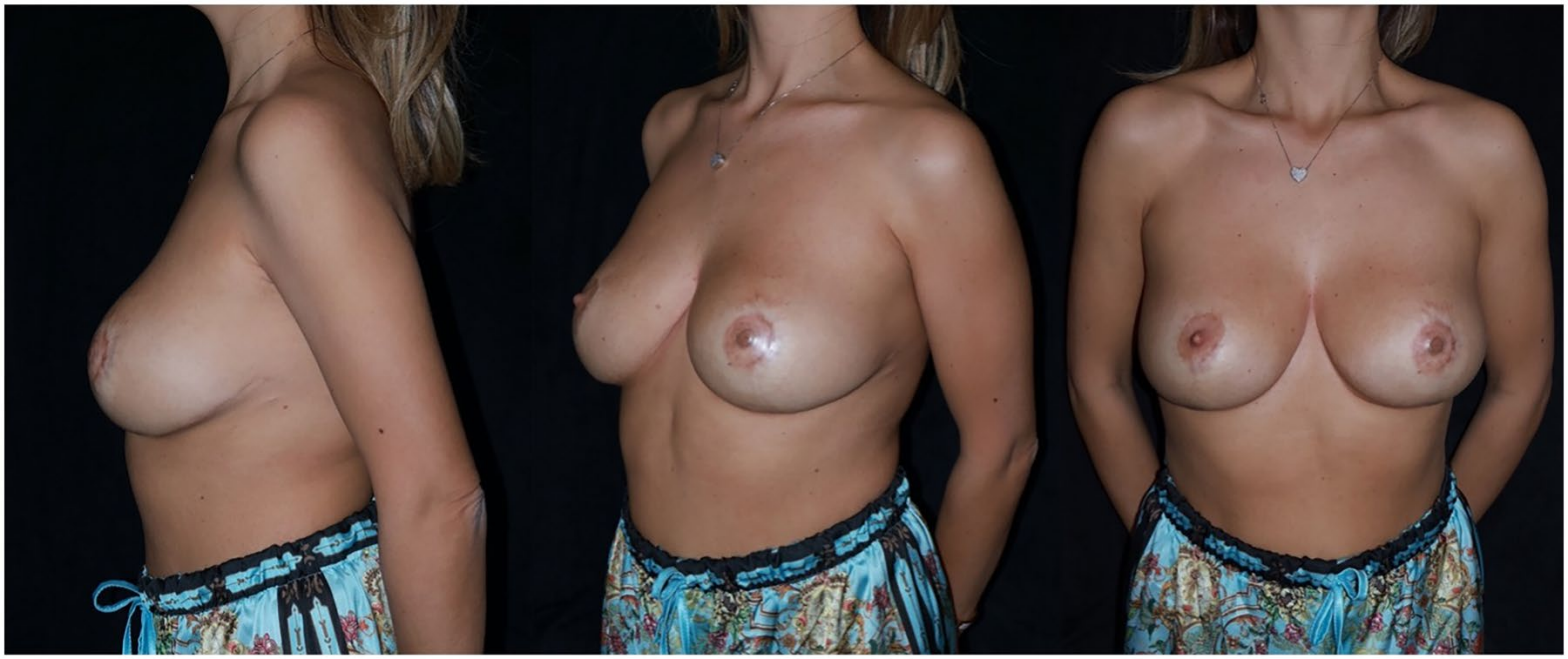
Postoperative photos showing the final result after FixNip implant positioning and 3d medical tattooing of the NAC.

**Figure 4.**
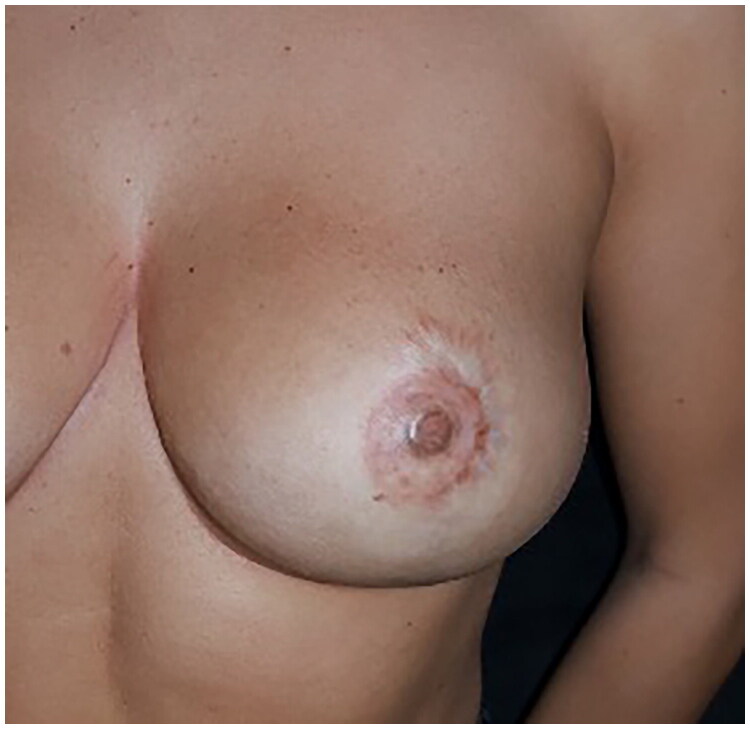
Close up photo of the final result.

Prior ultrasound evaluation for implant integration and positioning, 8 months later the patient undergoes medical tattooing of the areola. To date, two years have passed since FixNip prosthesis placement, with no reported complications, and the implant has remained well-positioned. Long-term ultrasound evaluation did not show a periprosthetic capsule of clinically relevant thickness, and the skin overlying the implant remained soft and pliable, without increased hardness or unnatural sensation around the NAC. No signs of tissue breakdown or implant exposure have been observed (Figures 3 and 4).

Both patient and her partner reported to perceive the implant beneath the skin; however, this did not cause discomfort, concern, or embarrassment during intimacy, and sexual activity was not affected.

## Discussion

The NAC is perhaps the most important aesthetic subunit and focal point of the normal breast. In patients undergoing breast reconstruction procedures for oncological reasons, NAC restoration may be seen as ‘the cherry on the cake’ in reconstruction procedures [12,13].

Different techniques have been proposed, including local flaps, autologous grafts (fat [14], cartilage [15], dermal [16]), allogeneic tissue (acellular dermal matrix [17], lyophilized allogeneic costal cartilage [18]), and synthetic materials (fillers [19] et al.). NAC tattooing has recently gained popularity with the introduction of the hyper realistic 3-D tattoos, nevertheless the absence of nipple projection and tattoo discoloration over time are responsible for patients’ dissatisfaction [20,21].

Total NAC necrosis following breast reduction is a very uncommon event, nevertheless it probably the most traumatic for both patient and surgeon. It is clear that, in these unfortunate circumstances, all the surgeon’s effort should be invested in reconstructing the lost NAC in the most perfect way. Although the patient did not exhibit the typical systemic high-risk profile for NAC necrosis (BMI 27, non-smoker, moderate resection weight), this case suggests that local vascular dynamics may play a more decisive role than systemic factors alone. In particular, the superomedial pedicle entails a relatively long arc of transposition, which may increase susceptibility to venous congestion, especially if subtle torsion or kinking occurs. Even in the absence of excessive tension at inset, minor alterations in venous outflow can progressively compromise perfusion. Postoperative external compression may have further contributed to impaired drainage. In this case, hematoma and infection were clinically excluded, supporting the hypothesis of a predominantly vascular–mechanical mechanism [22].

**Figure 5.**
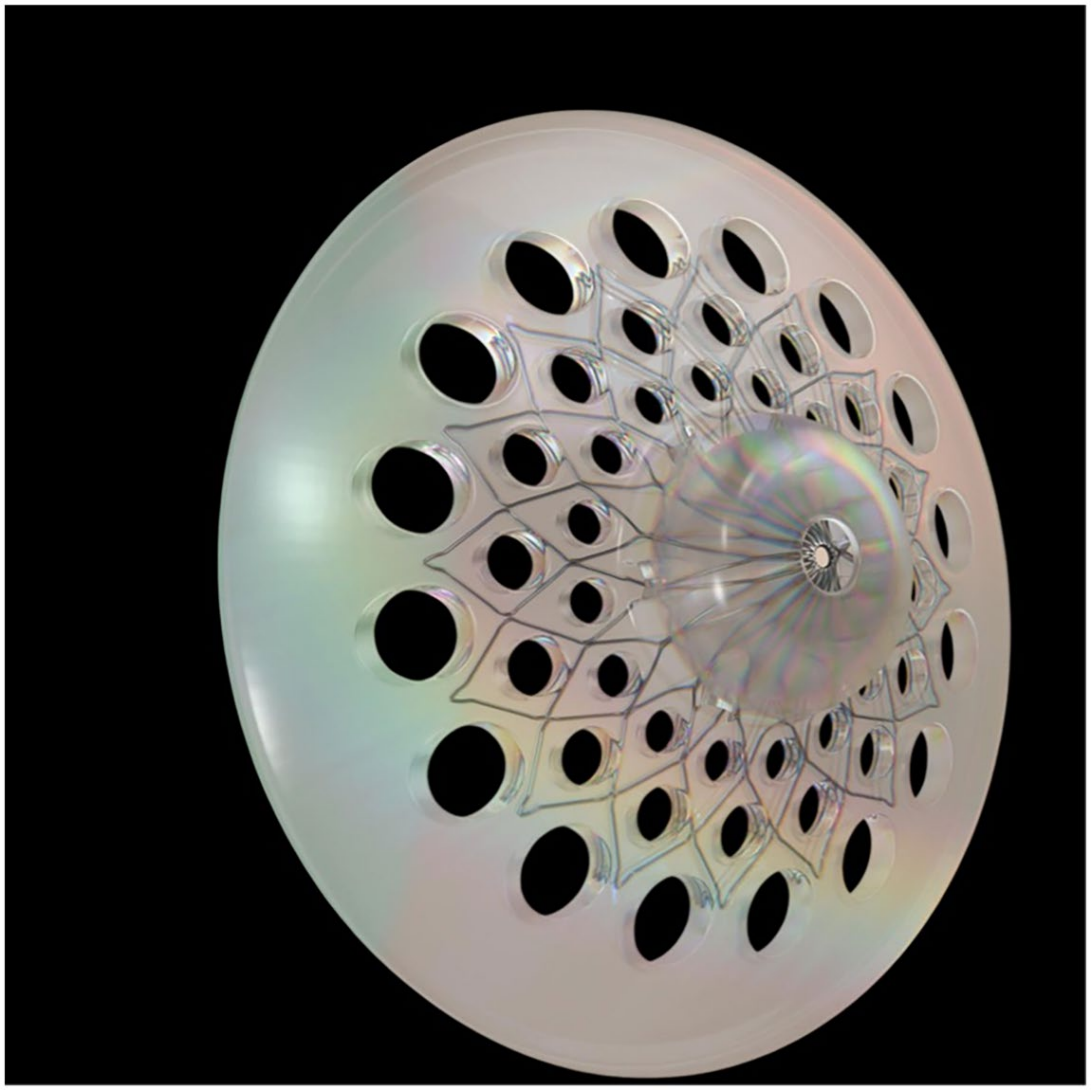
FixNip implant 3D structure.

The FixNip is a novel, implantable device developed by GC Aesthetics for nipple-areola complex reconstruction. It consists of a biocompatible silicone body supported by a floral-shaped nitinol (nickel-titanium alloy) frame. This internal scaffold enables the implant to be compressed for easy insertion through a minimal incision, and subsequently self-expands to its intended shape once *in situ*, providing immediate and durable nipple projection. The outer perforated silicone shell encourages soft tissue ingrowth, enhancing implant stability and minimizing the risk of migration or extrusion over time (Figure 5).

This newly developed NAC implant offers a unique, standardized approach to reconstruction and has demonstrated promising outcomes in oncologic breast surgery. Its placement can be performed in a short, minimally invasive outpatient procedure under local anesthesia. Clinical experience has shown good tolerability, minimal downtime, and a low complication profile.

One of the most compelling advantages of the FixNip implant is its ability to maintain long-term nipple projection—a key limitation of traditional autologous flap techniques, where flattening over time is a common cause of patient dissatisfaction [12]. Additionally, unlike tattoo-only reconstructions, the FixNip provides both three-dimensional form and tactile realism, improving patient-perceived naturalness and body image satisfaction. This makes the FixNip a particularly valuable option in both in oncologic reconstruction and salvage scenarios in cases of total NAC loss following breast reduction surgery. Nevertheless, while this device may represent a useful adjunct in selected cases, its use must be carefully considered in light of potential limitations and risks. As with any implanted foreign material, there is an inherent risk of infection, chronic inflammation, and device-related complications. In patients with thin or scarred soft tissues, the risk of palpability or exposure may be increased, particularly in areas subjected to tension or limited vascularity.

In addition, as the device is composed of nitinol (nickel–titanium alloy), hypersensitivity reactions, although rare, should be considered, especially in patients with known metal allergies. Preoperative assessment for a history of nickel sensitivity may therefore be advisable. Furthermore, should infection occur, management may require prolonged antibiotic therapy and, in refractory cases, device removal. Also, postoperative imaging follow-up should be used to monitor device position and tissue integration, particularly in the early phases of clinical adoption.

Finally, current evidence is limited, and larger studies with longer follow-up are necessary to better define safety, complication rates, and long-term outcomes.

Despite these encouraging results and the satisfactory long-term outcomes observed, it is important to recognize the limitations of this report. Being a single-patient case, generalizability is inherently limited. In addition, the prosthesis entails a financial cost, which may influence reconstructive choices, patient selection, and the wider adoption of this approach. Furthermore, patient satisfaction was assessed qualitatively based on the patient’s subjective report during follow-up, and no validated patient-reported outcome measures or structured satisfaction scales were administered. The absence of standardized tools, such as the BREAST-Q module, as well as objective measurements (e.g. nipple projection over time), represents a limitation of this report. Future studies should incorporate formal outcome measures to provide more robust and reproducible data.

## Conclusions

Given the psychological impact associated with NAC loss, especially in young or otherwise healthy patients undergoing elective procedures such as breast reduction, FixNip offers an emotionally and technically satisfying reconstructive alternative. As such, it represents a meaningful advancement in the repertoire of techniques available to plastic surgeons aiming to restore not just anatomy, but confidence and identity.
